# Annual Editorial – Best Videos of the Year in 2025

**DOI:** 10.1590/S1677-5538.IBJU.2026.01.03

**Published:** 2026-02-02

**Authors:** Philippe E. Spiess

**Affiliations:** 1 Department of GU Oncology Moffitt Cancer Center FL United States Department of GU Oncology Moffitt Cancer Center, FL, United States; 2 Department of Tumor Biology Moffitt Cancer Center FL United States Department of Tumor Biology Moffitt Cancer Center, FL, United States

Dear readers,

As we close another remarkable year, I would like to extend my deepest gratitude to all of our readers, contributors, and our editorial team members who have made the International Brazilian Journal of Urology one of the most respected and recognized journals in our field. The video section has yet again truly thrived in recent years, thanks to the incredibly innovative submissions from colleagues around the world. These contributions highlight the dedication and creativity of urologists who continue to advance the outcomes for our patients by the unrelenting commitment for setting new standards of quality and improved outcomes.

When selecting the "Best Videos of the Year," several important factors were carefully considered: innovation, the ability to enhance patient care, and clarity in demonstrating techniques that colleagues can integrate into their own practice. It is with great pleasure that I now announce this year's top three selections.

## First Prize – Best Video of the Year

Robotic Bilateral Ileal Interposition: Total Intracorporeal Approach

Authored by Dr. Chi-Hang Yee and colleagues in the Department of Surgery, Prince of Wales Hospital, Hong Kong (e20250359; Issue 6, 2025)

This video represents a landmark achievement in the management of severe ureteral stricture disease associated with ketamine-related nephropathy. The team elegantly demonstrates a minimally invasive robotic approach of ileal interposition using a total intracorporeal approach. The procedure is not only technically impressive but also provides hope for patients with highly complex pathology such as this to be offered such surgical approaches. We congratulate this group for their extraordinary innovation and commitment to advancing minimally invasive surgery in such challenging scenarios.

## Second Prize

Robotic-Assisted Laparoscopic Ureterocalicostomy (RALUC): How we do it

Authored by Dr. Stolzenburg and colleagues from institutions in Germany, Greece, and Brazil (e20250125; Issue 5, 2025)

This beautifully constructed video provides a meticulous, step-by-step depiction of robotic-assisted laparoscopic ureterocalicostostomy for complex upper urinary tract strictures and/or obstruction. Such procedures are rarely performed in day-to-day clinical practice, and the lack of high-quality educational resources are readily available for colleagues. This contribution addresses that gap, offering colleagues around the world a reliable, consistent, and minimally invasive approach with anticipated excellent patient outcomes. We commend this team for their dedication to surgical education and technical excellence.

## Third Prize

Mixed Reality Ultrasound-Guided Mini-ECIRS with Apple Vision Pro©: First Case Report

Authored by Dr. Montoro Neto and colleagues, from several institutions in Sao Paulo, Brazil (e20240610; Issue 2, 2025)

This pioneering work highlights the integration of emerging technology—mixed reality with the Apple Vision Pro—into endourological practice. The video demonstrates how advanced interface tools can support surgeons in performing complex procedures with enhanced visualization and accessibility. This case underscores the innovative spirit of our field and the forward-looking approach of our contributors.

In closing, I wish to express my sincere appreciation to our Editor-in-Chief Dr Luciano Favorito and the entire editorial team for their tireless efforts in maintaining the highest standards of academic and publishing excellence. It is with pride that we see our journal continuing to grow in reputation and influence within the global urological and scientific community. On behalf of our team, I extend my warmest wishes for the holiday season and for a healthy, prosperous, and fulfilling year ahead.
